# Addressing muscle–tendon imbalances in adult male athletes with personalized exercise prescription based on tendon strain

**DOI:** 10.1007/s00421-024-05525-z

**Published:** 2024-06-06

**Authors:** Kolja Weidlich, Theresa Domroes, Sebastian Bohm, Adamantios Arampatzis, Falk Mersmann

**Affiliations:** 1 Department of Training and Movement Sciences, Humboldt-Universität zu Berlin, Philippstr. 13, Haus 11, 10115 Berlin, Germany; 2 Berlin School of Movement Science, Humboldt-Universität zu Berlin, Berlin, Germany

**Keywords:** Patellar tendon adaptation, Plyometric loading, Prevention, Tendon strain, Individualization, Muscle–tendon diagnostics

## Abstract

**Purpose:**

Imbalances of muscle strength and tendon stiffness can increase the operating strain of tendons and risk of injury. Here, we used a new approach to identify muscle–tendon imbalances and personalize exercise prescription based on tendon strain during maximum voluntary contractions (ε_max_) to mitigate musculotendinous imbalances in male adult volleyball athletes.

**Methods:**

Four times over a season, we measured knee extensor strength and patellar tendon mechanical properties using dynamometry and ultrasonography. Tendon micromorphology was evaluated through an ultrasound peak spatial frequency (PSF) analysis. While a control group (*n* = 12) continued their regular training, an intervention group (*n* = 10) performed exercises (3 × /week) with personalized loads to elicit tendon strains that promote tendon adaptation (i.e., 4.5–6.5%).

**Results:**

Based on a linear mixed model, ε_max_ increased significantly in the control group over the 9 months of observation (*p*_Con_ = 0.010), while there was no systematic change in the intervention group (*p*_Int_ = 0.575). The model residuals of ε_max_, as a measure of imbalances in muscle–tendon adaptation, demonstrated a significant reduction over time exclusively in the intervention group (*p*_Int_ = 0.007). While knee extensor muscle strength increased in both groups by ~ 8% (*p*_Con_ < 0.001, *p*_Int_ = 0.064), only the intervention group showed a trend toward increased normalized tendon stiffness (*p*_Con_ = 0.824, *p*_Int_ = 0.051). PSF values did not change significantly in either group (*p* > 0.05).

**Conclusion:**

These results suggest that personalized exercise prescription can reduce muscle–tendon imbalances in athletes and could provide new opportunities for tendon injury prevention.

## Introduction

A key factor for movement performance is the coordinated interaction between muscle and tendon. Tendons transmit the forces generated by the muscle to the skeleton and enable the storage and release of elastic strain energy during movement. Their elasticity affects the muscle’s operating length and velocity, modulating its force potential and enthalpy efficiency (Bohm et al. 2021; Nikolaidou et al. 2017; Kawakami and Fukunaga 2006; Roberts 2016). The efficacy of this energy exchange seems to be particularly high when the stiffness of a tendon is geared to the strength capacity of its muscle (Lichtwark and Wilson 2007; Mendoza and Azizi 2021; Orselli et al. 2017) and the neuromuscular activation is precisely timed during movement (Sawicki et al. 2015). Consequently, a training-induced gain in muscle strength may not translate effectively into movement performance, when the finely tuned balance of the muscle–tendon unit is disrupted due to a deficit in tendon compared to muscle adaptation.

An increase in tendon operating strain due to an imbalanced muscle and tendon adaptation may also increase the risk of tendon injury. Though the precise determination of ultimate tendon strain in vivo is not possible in humans, there appears to exist an inherent consistency in ultimate strain across varying tendons and species (LaCroix et al. 2013). This implies that a higher tendon operating strain leads to an increased mechanical demand for the tissue due to the higher ratio of operating strain to ultimate strain (Alexander 1981). It has been demonstrated in vitro that the initial tendon strain during both static and cyclic loading determines the time or number of cycles to tissue failure (Wren et al. 2003). A study on rabbits’ Achilles tendons further illustrated that catabolic processes and tissue damage outweigh the tissue’s repair mechanisms at tendon strains of 9% during cyclic loading (Wang et al. 2013). In fact, several longitudinal and cross-sectional studies reported notably high levels of tendon strain and elevated strain fluctuations in athletes from several sports with a high prevalence of tendinopathy (Charcharis et al. 2019; Karamanidis and Epro 2020; Pentidis et al. 2021), particularly in volleyball athletes (Mersmann et al. 2016; 2017). There is also first in vivo evidence in male adolescent athletes that high levels of strain may induce localized disorganization within the collagen structure of tendons (Mersmann et al. 2019; 2021). Additionally, a recent prospective longitudinal study from our group demonstrated that adolescent athletes with tendon strains of ≥ 9% during maximum effort muscle contractions have a 2.3-fold elevated risk of developing tendon pain (Mersmann et al. 2023). Therefore, we can argue that interventions that promote a balanced adaptation of muscle strength and tendon stiffness and prevent high levels of tendon strain could make an important contribution to reducing tendon injury risk in certain athletic populations.

In 2020 (Arampatzis et al. 2020), we introduced an approach that uses the maximum tendon strain during a maximal voluntary isometric contraction to identify individual muscle–tendon imbalances in terms of deficits in either muscle strength (i.e., low strains) or tendon stiffness (i.e., high strains), and derive respective exercise recommendations. Muscle strength deficits may be addressed with loading modalities that seem to provide a more effective stimulus for muscle than tendon adaptation (e.g., moderate intensity loading until failure), while targeted improvements in tendon stiffness may be achieved by using personalized loads corresponding to a certain range of tendon strain. In their 'homeostatic calibration point’ theory, Lavagnino and Arnoczky (Lavagnino and Arnoczky 2005; Lavagnino et al. 2006) proposed the existence of upper and lower bounds of tendon strain that result in a net anabolic response. The theory found support in animal models (Wang et al. 2013, 2015) and led to the conception of a beneficial strain range (‘sweet spot’) for enhancing the tendon’s mechanical and morphological properties (Pizzolato et al. 2018; Docking and Cook 2019). Through a series of systematic intervention studies conducted in vivo, we have established that cyclic loading of human tendons within a 4.5–6.5% strain range effectively promotes increases in tendon stiffness, mainly via an improvement in its material properties (Arampatzis et al. 2007, 2010; Bohm et al. 2014). This tendon strain range was achieved at approximately 90% of an isometric maximum voluntary contraction (MVC). However, in athletes with marked deficits in tendon stiffness (i.e., high tendon strain during an MVC), this strain range is reached already at rather low loads, which may provide the opportunity to achieve a comparatively stronger stimulus for the tendon if the mechanical and metabolic stress for the muscle is low.

Recently, we applied the proposed approach for the identification of muscle–tendon imbalances and personalized exercise prescription to a sample of adolescent handball athletes (Domroes et al. 2024). The intervention led to a clear reduction of muscle–tendon imbalances and particularly promoted an increase in tendon stiffness in athletes with high tendon strains at baseline. However, there seems to be a significant reduction in tendon collagen turnover from adolescence to adulthood (Heinemeier et al. 2013) and the number of athletes developing deficits in tendon stiffness seems higher in adults compared to adolescents (Charcharis et al. 2019). Due to the high prevalence of both musculotendinous imbalances and tendinopathy in adult volleyball athletes (Charcharis et al. 2019; Kulig et al. 2013; Lian et al. 2005), the purpose of the present study was to examine the effect of a personalized exercise prescription based on the individual maximum tendon strain, on the adaptation of the patellar tendon, muscle–tendon imbalances, and changes in tendon micromorphological structure in this population. We hypothesized that: (a) A personalized training program would promote a balanced knee extensor muscle and patellar tendon adaptation over the course of a competitive season, reduce fluctuations in patellar tendon strain and, thus, reduce the frequency of elevated maximum tendon strains  ≥ 9%. (b) This personalized approach to training would prevent structural disorganization of the tendon tissue that has been associated with high tendon strain exposure and tendinopathy (Kulig et al. 2013; Mersmann et al. 2019).

## Materials and methods

### Participants and experimental design

With regard to the design of the study, two groups of adult male volleyball players needed to be recruited: an intervention group that would receive a personalized muscle–tendon training program and a control group that would adhere to their usual training routine. A power analysis was conducted in G*Power (version 3.1.6; HHU, Düsseldorf, Germany) before recruitment to estimate the necessary sample size per group. In an earlier study, we found fluctuations of maximum tendon strain (as an indicator for imbalances in muscle–tendon adaptation) of 0.86 ± 0.40% in adolescent volleyball athletes and 0.31 ± 0.14% in untrained controls (i.e., Cohen’s *d* = 1.8; Mersmann et al. 2016). We assumed that the personalized intervention could reduce the fluctuations of tendon strain by about 40% (or 0.34 percentage points), which results in an effect size of *d* = 1.2 for the comparison between athletes of the intervention group and the controls. For a power of 0.8, a sample size of *n* = 12 per group was calculated. Considering a potential drop-out, 13 and 15 participants were recruited for the control and intervention group, respectively, from two teams from the same division (3rd German Volleyball league). While the players of one team served as control group, the other received the personalized intervention. The sport-specific loading in both groups consisted of an average of two regular training days (2 h each), one competition day (4 h), and one additional athletic training session (1.5 h, focusing on core strengthening and athletic abilities) per week. Participants were excluded if they had neurological or musculoskeletal impairments, while those with patellar tendon pain were included if they could perform maximum voluntary muscle strength testing. Four times over a competitive season (M1–4; M1: one month into the season, M4: one to two month into the off-season), the maximum isometric knee extensor strength, patellar tendon stiffness, and maximum tendon strain were determined using inverse dynamics and ultrasonography. Additionally, we assessed changes in the micromorphological structure of the tendon and the prevalence of tendon pain. Due to scheduling conflicts (e.g., tournaments, sickness, vacation), the time intervals between measurements differed between groups. In the intervention group, the intervals were approximately 17, 12, and 6 weeks between measurements, while the control group had a uniform 12-week interval between measurements. The differing intervals were considered in our analysis (see statistics section). The time between the first measurement and the initiation of the personalized training program was 2.7 ± 0.8 weeks. The participants gave their written informed consent for the experimental procedures, which were approved by the Ethics Committee of the Humboldt-Universität zu Berlin (HU-KSBF-EK_2020_0005) and followed the standards of the Declaration of Helsinki. All measurements were performed on the dominant leg, which was defined as the leg used to kick a ball. The prevalence of patellar tendon-related pain and functional limitations was assessed using the validated German version of the VISA-P questionnaire, which considers symptoms occurring over the past two weeks (Lohrer and Nauck 2011). Participants scoring 87 points or lower were classified as symptomatic, as this score signifies the minimum clinically important difference from the questionnaire's maximum score of 100 points (Lohrer and Nauck 2011). Participants in both groups were excluded if they were not able to attend at least three of the four main measurements. Furthermore, participants of the intervention group who failed to achieve an average training frequency with the personalized exercises of at least two times per week were also excluded. In the intervention group, one participant was excluded from the study due to an injury unrelated to the intervention, two participants did not meet the required training frequency, and two participants withdrew voluntarily before completing the study for undisclosed reasons. In the control group, one participant was unable to complete the study due to an injury unrelated to the study. Therefore, the final sample size in the two groups was 12 and 10 athletes in the control and intervention group, respectively. In the control group, one athlete each in M1 and M4, and two in M3 were unable to attend the measurements.

### Assessment of maximum knee extensor muscle strength

**Fig. 1:**
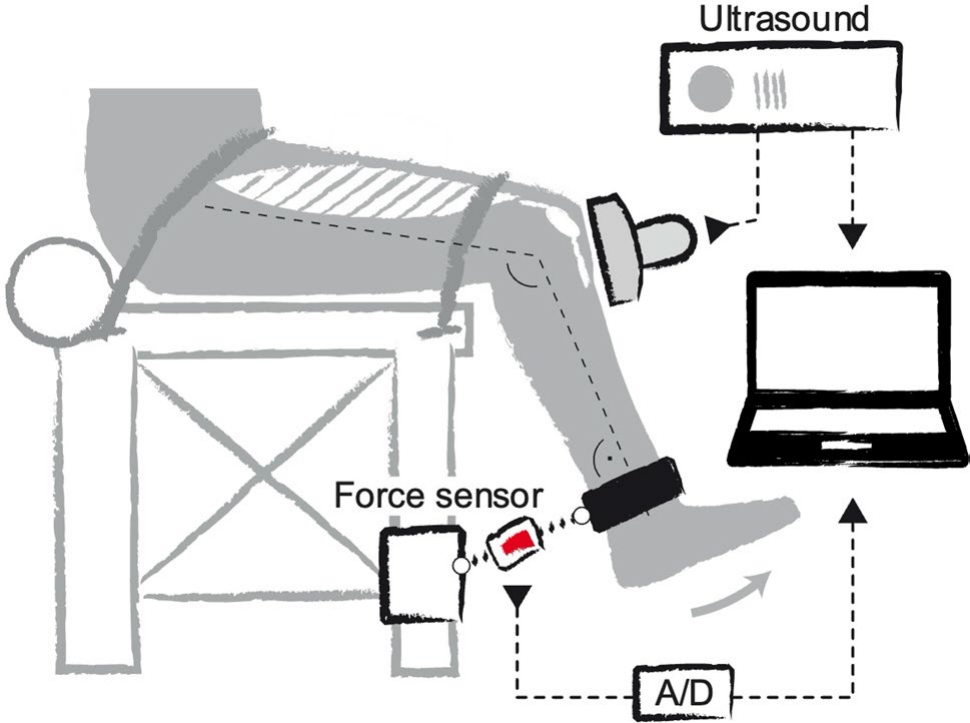
Mobile measurement system. The force generated during knee extensions at 60° knee joint angle (0° represents full knee extension) was recorded with a force sensor, which was oriented perpendicular to the shank. To calculate the forces exerted on the tendon, the measured forces were multiplied by the external lever arm of the applied force—specifically, the distance from the lateral epicondyle to the center of the strap attached to the shank—then corrected for gravitational moments and divided by the patellar tendon moment arm, determined through anthropometry. The elongation of the patellar tendon during contractions was recorded synchronously using ultrasound

After a standardized warm-up, which included 10 submaximal isometric knee extension contractions with increasing intensity to precondition the tendon (Maganaris 2003) and accustom the participants to the experimental situation, the participants performed two MVCs on a mobile diagnostic device (Fig. 1) at a knee angle of 60° during the plateau of the contraction (0° representing full extension; measured using a goniometer with the assumption of the axis of rotation at the lateral femoral condyle; Churchill et al. 1998) and the trunk in an upright position. This knee joint angle was selected because it is the approximate optimum for knee extensor muscle force generation with regard to the force–length relationship (Herzog et al. 1990). A third trial was performed in case there was an increase from the first to the second trial of  > 5%. A strap was fixed around the shank, perpendicularly connected with a non-elastic rope to a force sensor (2 kN; Biovision, Wehrheim, Germany). The applied forces were recorded at 200 Hz, then low-pass filtered with a second-order Butterworth filter and a 6 Hz cut-off frequency, using a custom-written MATLAB interface (version R2016a, MathWorks, Natick, USA). The maximum force value was determined based on a moving average with a time window of 50 ms. Resultant knee joint moments were calculated as the product of the force recorded during the MVC and the distance of the point of force application on the center of the strap to the lateral femoral condyle as the assumed location of the axis of rotation (Churchill et al. 1998). The moments were corrected for the moments of gravity of the shank and foot segment based on the participants’ anthropometry (Dempster 1955). The highest moment of the MVC trials represented the maximum strength of the knee extensors and was used for further analysis (both absolute moments and moments normalized to body mass).

### Assessment of the patellar tendon force–elongation relationship and maximum strain

To determine the force–length relationship of the patellar tendon, five ramp contractions were performed in the setup described above, where the generated force was steadily increased from rest to 90% of the MVC within ~ 5 s. The participants received visual feedback of the target and generated force during the ramp contractions. The elongation of the patellar tendon during these isometric contractions was recorded using a 10 cm linear ultrasound probe (MyLab60; Esaote, Genoa, Italy; probe: LA923, 7.5 MHz; 25 Hz image frequency). For this purpose, the probe was fixed over the patellar tendon with a modified knee brace, mediolaterally centered and perpendicular to its longitudinal axis. In each trial, a manually triggered 5 Volt signal was simultaneously dispatched to both the MATLAB interface for force recording and the ultrasound device to synchronize the data during the subsequent analysis. For measuring tendon elongation, we quantified the displacement of the deep insertion sites of the tendon at the patella and at the tibial tuberosity, which was tracked with a semi-automatic software (Tracker Video Analysis and Modeling Tool V.4.92, Open Source Physics, Aptos, California, USA). For every trial, the tendon resting length was measured in the resting state prior to the contraction at best possible relaxation and equilibrium of moments, and then averaged over all trials. The slack of the tendon at rest was taken into account by calculating tendon resting length using a spline fit through the lower insertion points of the tendon and two additional markers along the deep peritendinous sheath (Mersmann et al. 2017). The maximum force applied to the tendon (i.e., maximum tendon force; TF_max_) was determined based on the highest MVC trial of each participant. It was calculated by dividing the respective knee joint moment by the tendon lever arm, which was predicted based on anthropometric data (Mersmann et al. 2016) and adjusted to the knee angle of 60° (Herzog and Read 1993). The patellar tendon force–elongation relationship for each single ramp trial was saved as a piecewise-linear polynomial function in MATLAB (version R2019b; ‘interp1’ function with ‘pp’ option), using every measured data point of each ramp as input. To achieve a high reliability (≥ 0.95) and observer independence (Schulze et al. 2012) we averaged the five single force–elongation curves. Therefore, at each measured data point, all single ramp trials were evaluated using their respective piecewise-linear polynomials and then averaged (‘fncmb’ function in MATLAB). Then, on the averaged patellar tendon force–elongation curve, a second-order polynomial fit passing through zero (MATLAB function ‘polyfitZero’, version 1.1) was applied to obtain the final patellar tendon force–elongation curve for each participant. Patellar tendon stiffness was calculated as the quotient between 50 and 85% of TF_max_ from the polynomial function. Normalized patellar tendon stiffness was determined by multiplying tendon stiffness by its resting length (and reported in kN/strain), effectively representing the slope of the tendon force–strain curve, which is more robust against variations in tendon length. Maximum tendon strain was determined by extrapolating the tendon elongation to TF_max_, using the polynomial fit of the force–elongation curve, and then dividing the maximum elongation by the tendon’s resting length.

### Assessment of patellar tendon micromorphology

The micromorphology of the tendon was estimated based on a spatial frequency analysis of ultrasound images as suggested by Bashford et al. (2008) of the proximal part of the patellar tendon (Mersmann et al. 2019). The assessment was conducted prior to the measurement of muscle strength and the tendon force–elongation relationship to minimize potential effects of acute tendon loading (Drongelen et al. 2007; Rosengarten et al. 2015). To remove tendon slack, yet not applying substantial forces to the tendon, participants were placed in a supine position with a 90° knee angle. A linear probe of the ultrasound system (LA523, 13 MHz, depth: 3.0 cm) was positioned over the patellar tendon parallel to its longitudinal axis and below the distal end of the apex patellae. Two sequences were recorded, and the images were analyzed using a custom MATLAB interface. A polygon, covering a length of 40% of the tendon’s resting length and the full thickness of the tendon, extending from the inferior insertion at the patellar apex to the midportion of the tendon was defined as region of interest (ROI). The maximum possible number of 32 × 32 pixel kernels within the ROI were analyzed by applying a 2D Fast Fourier Transform, followed by a high-pass filter with a radial frequency response and a half-power cut-off frequency of 1.23 mm^−1^. The filtered kernels were zero-padded in both dimensions to a size of 128 × 128 pixels and the average distance from the peak spatial frequency (PSF) of all kernels to the spectral origin in the frequency spectrum was used to evaluate the packing density and orientation of the collagen bundles. Low PSF values correspond to a less compact and isotropic speckle pattern in the ultrasound images, which is characteristic of tendinopathic tendons (Kulig et al. 2013) and may also be a consequence of high tendon strain (i.e., mechanical demand) in asymptomatic tendons (Mersmann et al. 2019, 2021). The average of the two recorded trials per participant was used for statistical analysis.

### Personalized exercise prescription

The intervention group underwent a personalized exercise program throughout the competitive season, with the objective of promoting a balanced muscle–tendon adaptation. Dependent on the individual’s maximum patellar tendon strain, the loading program for the subsequent exercise phase (~ six weeks until the next diagnostic assessment) was selected as follows: If the maximum tendon strain (ε_max_) was below 4.5%, it was interpreted as an indication that the muscle had a strength deficit relative to the stiffness of the tendon. Consequently, the intention was to schedule a training that is considered more effective in increasing muscle strength than tendon stiffness. For example, performing medium-load contractions until muscle failure provides an effective stimulus for muscle hypertrophy (Mitchell et al. 2012; Burd et al. 2010), but does not promote tendon adaptation due to the low levels of strain during exercise and, thus, can induce a disproportionate increase of muscle strength compared to tendon stiffness (Lambrianides et al. 2024). However, none of the participants met this criterion at any time point during the intervention. Patellar tendon strains in athletes that fell within the intermediate range of 4.5 ≤ ${\upvarepsilon }_{{{\text{max}}}}$ < 9% were classified as having a balanced relationship between muscle strength and tendon stiffness, while strains ≥ 9% indicated a relative deficit in tendon stiffness compared to muscle strength. In both cases, an isometric loading program was prescribed with a personalized relative load corresponding to 5.5% of tendon strain, as this falls within the range of strain effective for tendon adaptation (Arampatzis et al. 2007, 2010; Bohm et al. 2014). The relative load was calculated based on the assumption of a linear relationship between tendon force and strain, and limited to a maximum of 90% MVC (i.e., in participants with maximum strains between 4.5 and 6.1%), to ensure a sufficient number of loading cycles could be realized during training. Consequently, the prescribed relative load for the target tendon strains was low in athletes with high levels of maximum strain, which was thought to provide a stronger stimulus for the tendon compared to muscle when the loading volume and, thus, the level of muscular fatigue were low.

**Fig. 2:**
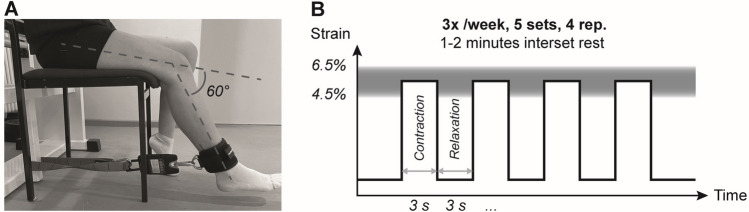
Training setup (**A**) and loading protocol (**B**) for the personalized tendon exercises. Isometric knee extensions were performed at 60° knee joint angle, with 0° representing full knee extension. A digital scale, attached to an adjustable non-elastic band, monitored the applied load. The personalized training load was derived individually from an MVC performed in the training device (updated every 2 weeks) and the relative load determined based on tendon strain determined at the main measurement time points and respective intermediate measurements

The isometric loading protocols were conducted using mobile training devices, which were designed to closely resemble the experimental setup of the main measurements to achieve the best possible consistency between the tendon strains achieved at a given relative load in the two settings (i.e., measurement and training). Accordingly, the devices consisted of a length-adjustable rigid band in series with a load scale, which was attached to the participants’ shank and allowed for real-time feedback of the applied forces during isometric knee extension contractions, which were performed at a knee angle of 60° as in the diagnostics (Fig. 2A). By performing an MVC in this specific training device to account for potential differences in the positioning of the point of force application and the orientation of the load scale with regard to the shank, each participant's individual absolute training load in this setting corresponding to the prescribed relative loading intensity (i.e., %MVC) was determined. Based on previous systematic research (Arampatzis et al. 2007, 2010; Bohm et al. 2014), the loading protocol then consisted of five sets of four isometric contractions. Each contraction was maintained for three seconds, followed by a three-second relaxation phase (Fig. 2B). The exercises were not performed additionally, yet incorporated into the athletes’ existing training schedule (i.e., as component within the regular and athletic training sessions) three times per week and were conducted on both legs at the same relative load. Adjustments of the absolute load (i.e., determination of a new MVC) were implemented every two weeks using the mobile training devices to account for increases in muscle strength. Intermediate diagnostics of muscle strength and tendon mechanical properties were carried out in addition to the regular measurements six weeks after M1 and M2 (note that M3 and M4 were only 6 weeks apart in the intervention group), to adjust the relative exercise load intensity based on the maximum strain of the patellar tendon. The actual strains corresponding to the relative loads were calculated retrospectively based on the individual force–strain curves.

### Statistics

The statistical analyses were performed using R (v4.1.2, nlme package, R foundation for statistical computing, Vienna, Austria). A linear mixed-effects model (LMM) with restricted maximum likelihood estimation was applied to the investigated parameters. The standardized residuals were tested for normality using the Shapiro–Wilk test, and no violations were observed for any parameter. The LMM included the factor “group” (intervention and control) with group-specific y-intercept, slopes and variance of the residuals to examine time- and group-dependent developments. To account for variations in the time intervals between measurements across individuals and groups, the measurement time points were individually converted into weeks (from 0 to ~ 35 weeks.). Linear mixed-effects models are capable of handling missing data, which occurred in the present study due to participants not attending every measurement time point. The model equation was as follows:

$${y}_{ij}={\beta }_{0}+{\beta }_{1}{t}_{ij}+{g}_{i}{\beta }_{2}+{g}_{i}{\beta }_{3}{t}_{ij}+{b}_{0i}+{b}_{1i}{t}_{ij}+{r}_{ij}.$$

In this equation, $i$ indexes the participants, $j$ indexes the measurement sessions, ${g}_{i}$ is a binary variable representing the group (0 for control group, 1 for intervention group), ${t}_{ij}$ is the time in weeks of the $j$-th measurement for participant* i*, ${\beta }_{0}$ is the y-intercept constant for the control group, ${\beta }_{1}$ is the slope constant for the control group, ${\beta }_{2}$ is the difference in intercepts between the two groups, ${\beta }_{3}$ the difference in slopes between the two groups, ${b}_{0i}$ is the participant-specific y-intercept (random effect), ${b}_{1i}$ is the participant-specific slope (random effect), and ${r}_{ij}$ is the residual. As the terms involving ${g}_{i}$ are zero for the control group, ${\beta }_{0}$ and ${\beta }_{1}$ represent the intercept and slope for this group, respectively. For the intervention group, the intercept and slope are given by ${\beta }_{0}+{\beta }_{2}$ and ${\beta }_{1}+{\beta }_{3}$, respectively. This allows for testing the significance of changes over time in the control group (slope constant ${\beta }_{1}$), differences between groups at M1 (intercept constant ${\beta }_{2}$), and group differences in the time-dependent changes (slope constant ${\beta }_{3}$; henceforth referred to as *time-by-group interaction*). The intervention group was separately tested for the difference in the slope from zero. To identify the individual deviations from linear development (i.e., fluctuations) for the relevant parameters, we additionally tested the residuals of the LMM as absolute values with regard to effects of group, time and time-by-group interaction. The associations of maximum tendon strain at M1 to the changes in maximum tendon strain (i.e., M4–M1) and the relative changes of normalized stiffness (i.e., (M4–M1) ⋅ M1^−1^ ⋅ 100) over the season were examined using the Pearson correlation coefficient (*r*). The anthropometric parameters (age, body height and mass) at the initial measurement time point (M1) and training age (i.e., years of volleyball training) were compared between groups using a *t*-test for independent samples. Additionally, the frequency of VISA-P scores with ≤ 87 points as a classification for symptomatic tendons was documented (Lohrer & Nauck 2011). The alpha level was set at 0.05 for all statistical tests.

## Results

At M1, there were no statistically significant differences between the groups in age (intervention 27.5 ± 7.8 years, control 30.7 ± 1.9 years; *p* = 0.23; mean ± standard deviation), training age (intervention 13.4 ± 11.0 years, control 14.6 ± 5.2 years; *p* = 0.35), body height (intervention 1.87 ± 0.07 m, control 1.90 ± 0.08 m; *p* = 0.46), and mass (intervention 84.3 ± 7.7 kg, control 82.2 ± 7.9 kg; *p* = 0.55). In the intervention group, the relative intensity in the personalized exercises ranged from 42 to 90% MVC, with averages of 68 ± 18% for M1, 62 ± 12% for M2, and 62 ± 11% for M3. The corresponding average operating strains, retrospectively calculated based on the actual individual force–strain curves (not the linear approximation during prescription) were 6.3 ± 0.8%, 6.4 ± 0.9%, and 6.4 ± 0.7% for M1, M2, and M3, respectively.

**Fig. 3:**
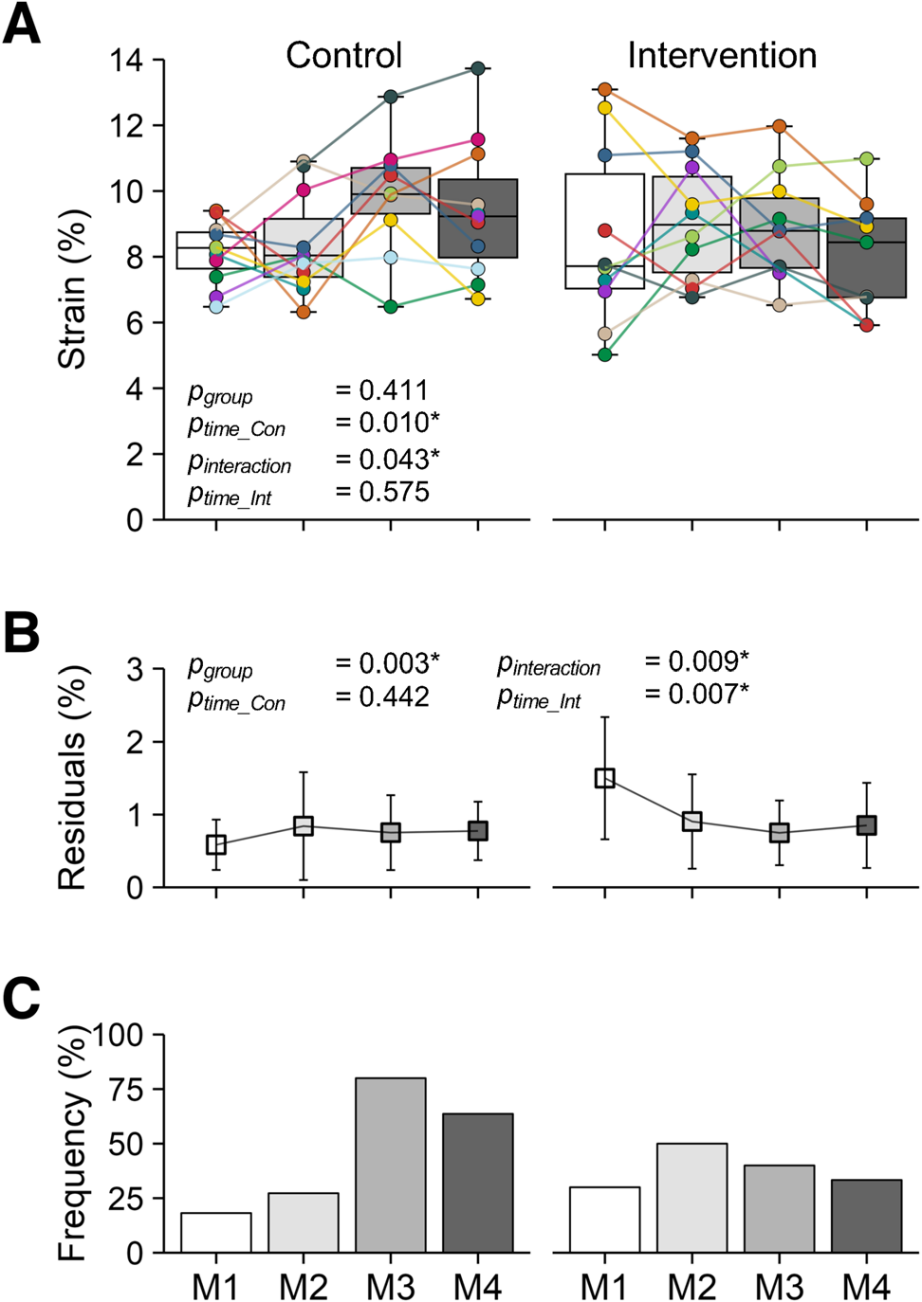
**A** Maximum patellar tendon strain, **B** Average residuals of the linear mixed model (mean ± SD) of tendon strain and **C** Frequency of athletes with high-level maximum patellar tendon strain (≥ 9%) in the control (left; *n* = 12) and intervention group (right; *n* = 10) at the four measurement sessions (M1–M4) throughout the season. Please note that, in the control group, one athlete each in M1 and M4, and two in M3 were not able to attend the measurements. In (**A**), the colored markers represent individual values and the median of the sample is shown within the boxes as horizontal line. The *p*-values refer to differences between groups at M1 (*p*_group_), the slope of the changes over time within the control (*p*_time_Con_) and intervention group (*p*_time_Int_), and time-by-group interactions (*p*_interaction_); *significant effect (*p* < 0.05)

The maximum patellar tendon strain did not differ significantly between the control and intervention group at M1 (*p* = 0.411). There was no significant change in the maximum tendon strain over time in the intervention group (*p* = 0.575), but there was a significant time-by-group interaction (*p* = 0.043) and a significant increase over time in the control group (*p* = 0.010, Fig. 3A). The residuals of maximum patellar tendon strain (as a measure of fluctuations; Fig. 3B) were significantly higher in the intervention group at M1 (*p* = 0.003). The residuals did not change significantly over time in the control group (*p* = 0.442), but there was a significant time-by-group interaction (*p* = 0.009) and a significant decrease of the residuals over time in the intervention group (*p* = 0.007, Fig. 3B). Descriptively, the frequency of athletes with high-level patellar tendon strain (i.e., ≥ 9%) increased in the control group (from 18% in M1 to 80% and 64% in M3 and M4, respectively) while no such increase over time was observed in the intervention group (38 ± 9% over all measurements; Fig. 3C).

**Fig. 4:**
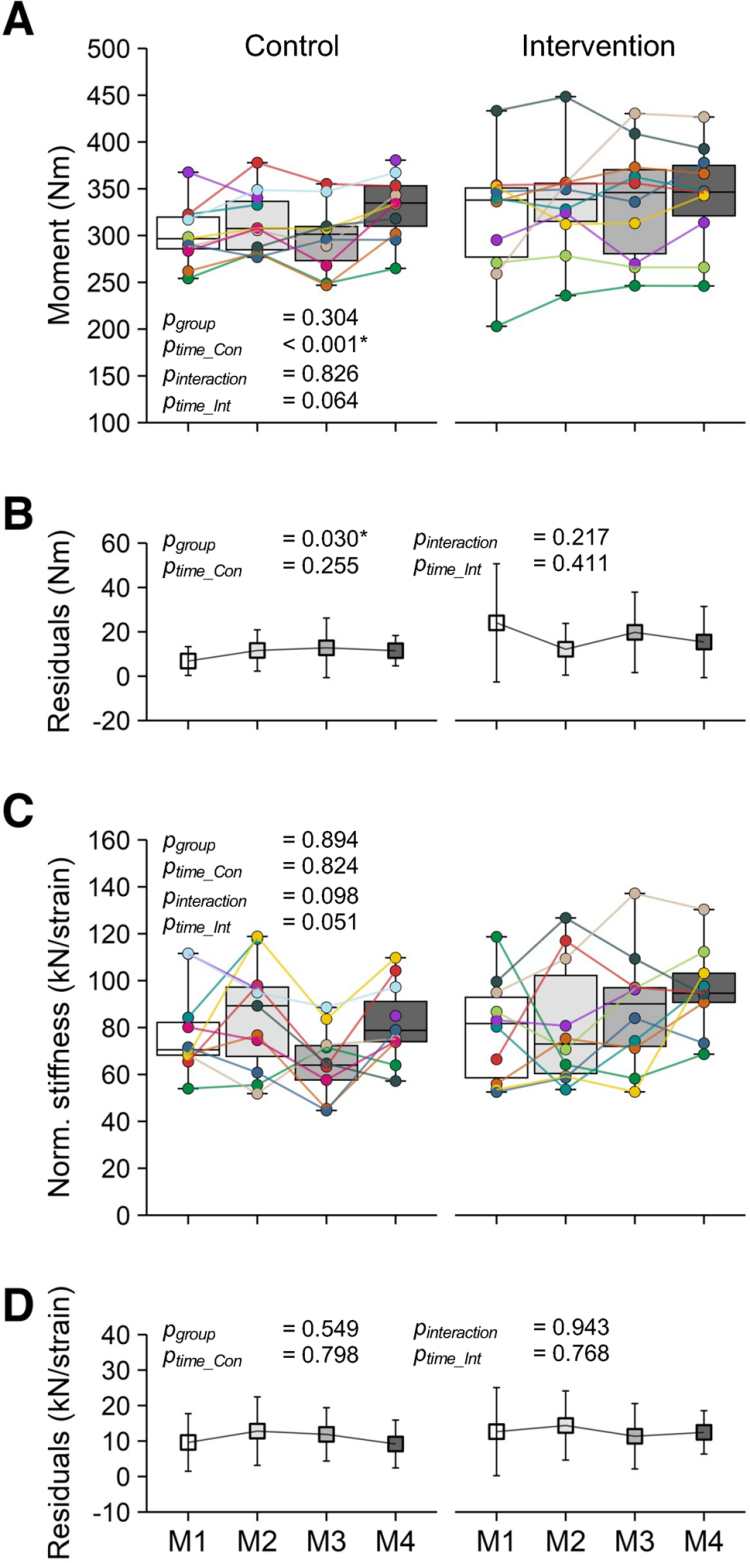
Maximum resultant knee joint moment (**A**) and normalized stiffness (**C**) in the control group (left; *n* = 12) and intervention group (right; *n* = 10) during a competitive season at four measurement time points (M1 to M4). Please note that, in the control group, one athlete each in M1 and M4, and two in M3 were not able to attend the measurements. The colored markers represent individual values, and the median of the sample is shown within the boxes (horizontal line). Below (**B**, **D**) are the respective average residuals of the linear mixed model (mean ± SD). The *p*-values refer to differences between groups at M1 (*p*_group_), the slope of the changes over time within the control (*p*_time_Con_) and intervention group (*p*_time_Int_), and time-by-group interactions (*p*_interaction_); *significant effect (*p* < 0.05)

The maximum knee joint moment (*p* = 0.304, Fig. 4A), the maximum knee joint moment normalized to body mass (*p* = 0.308, Table 1), and the maximum force applied to the patellar tendon (*p* = 0.233, Table 1) did not differ significantly between the control and the intervention group at M1. The maximum absolute and normalized knee joint moment as well as the maximum tendon force increased significantly over time in the control group (*p* < 0.001, *p* = 0.049, *p* = 0.026) but showed only a tendency toward an increase in the intervention group (*p* = 0.064, *p* = 0.065, *p* = 0.061). There was, however, no significant time-by-group interaction (*p* = 0.826, *p* = 0.982, *p* = 0.900, Fig. 4A and Table 1). The residuals of the maximum knee joint moments were significantly higher in the intervention group at M1 (*p* = 0.030) but did not show significant effects of time in the control (*p* = 0.255) or intervention group (*p* = 0.411) and no significant time-by-group interaction (*p* = 0.217; Fig. 4B).

**Table 1:** Resultant knee extension moment normalized to body mass as well as patellar tendon stiffness, elongation, proximal patellar tendon peak spatial frequency (PSF), patellar tendon resting length and lever arm, and number of athletes reporting patellar tendon symptoms (VISA-P score ≤ 87) at the four measurement time points (M1–M4)

	Control			Intervention				
	M1 *n* = 11	M2 *n* = 11	M3 *n* = 10	M4 *n* = 11	M1 *n* = 10	M2 *n* = 10	M3 *n* = 10	M4 *n* = 10
Norm. moment (Nm/kg)*	3.66 ± 0.2	3.78 ± 0.3	3.68 ± 0.4	3.96 ± 0.3	3.83 ± 0.6	3.96 ± 0.4	4.02 ± 0.5	4.09 ± 0.5
Tendon force (kN)*	5.02 ± 0.48	5.24 ± 0.48	5.01 ± 0.56	5.54 ± 0.49	5.38 ± 1.02	5.60 ± 0.86	5.70 ± 0.95	5.77 ± 0.91
Stiffness (kN/mm)	1.51 ± 0.49	1.65 ± 0.46	1.31 ± 0.28	1.60 ± 0.30	1.56 ± 0.39	1.63 ± 0.45	1.60 ± 0.53	1.74 ± 0.56
Elongation (mm)*	4.2 ± 0.4	4.3 ± 0.7	4.9 ± 0.7	4.7 ± 0.7	4.4 ± 1.4	4.6 ± 1.1	4.7 ± 1.1	4.1 ± 1.1
PSF (mm^−1^)	1.65 ± 0.2	1.73 ± 0.1	1.70 ± 0.3	1.73 ± 0.1	1.78 ± 0.1	1.79 ± 0.1	1.81 ± 0.1	1.69 ± 0.2
Resting length (mm)	51.8 ± 5.1	51.8 ± 4.9	50.2 ± 6.7	51.1 ± 5.5	51.1 ± 5.9	50.9 ± 6.2	51.0 ± 6.0	50.3 ± 5.6
Lever arm (mm)	59.7 ± 1.5	59.7 ± 1.6	59.4 ± 1.6	59.8 ± 1.5	59.3 ± 1.6	59.3 ± 1.6	58.7 ± 2.8	59.3 ± 1.6
Symptomatic athletes	1	1	3	1	3	3	1	2

Means ± standard deviation of the given experimental data

*Significant effect of time in the control group (*p* < 0.05)

In both patellar tendon stiffness (Table 1) and normalized patellar tendon stiffness (Fig. 4C), there were no significant differences between the groups at M1 (*p* = 0.912 and *p* = 0.894) and no significant changes over time in the control (*p* = 0.919, *p* = 0.824) or intervention (*p* = 0.187, *p* = 0.051) group and no time-by-group interaction (*p* = 0.284, *p* = 0.098). The residuals of normalized stiffness did not differ significantly between groups at M1 (*p* = 0.549), and showed no significant changes over time in the control (*p* = 0.798) or intervention group (*p* = 0.768) and no time-by-group interaction (*p* = 0.943; Fig. 4D).

**Fig. 5:**
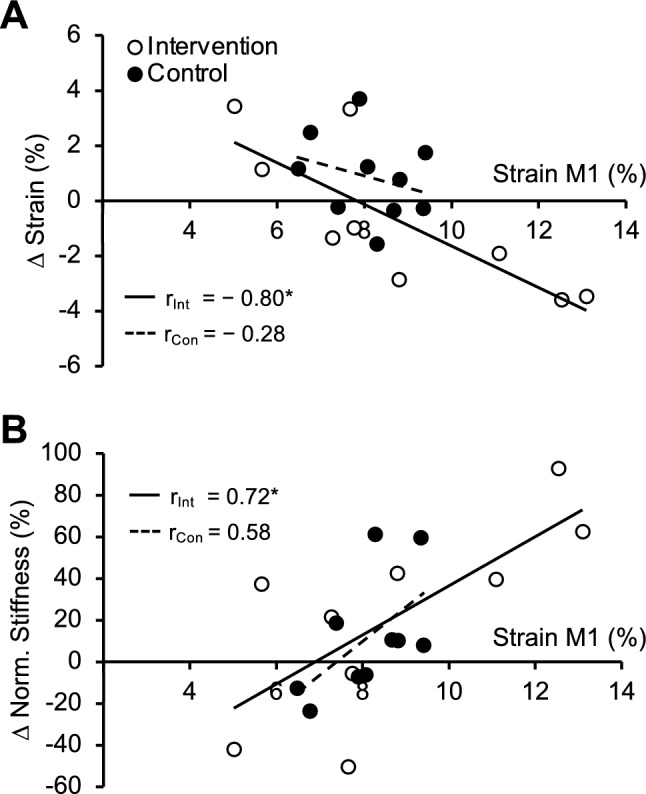
Association of maximum patellar tendon strain at baseline (M1) to (**A**) the change of maximum strain (in percentage points) and (**B**) the relative change of normalized tendon stiffness (in percent) from the first to the last measurement time point (M1–M4) for the control (black, dashed line; *n* = 10) and intervention group (white, solid line; *n* = 10). *r* Pearson correlation coefficient; *significant association (*p* < 0.05)

There was a significant negative correlation between maximum patellar tendon strain at M1 and changes in maximum patellar tendon strain from M1 to M4 in the intervention group (*r* =  − 0.80, *p* = 0.009) but not in the control group (*r* =  − 0.28, *p* = 0.428; Fig. 5A). Further, there was a significant correlation between maximum patellar tendon strain at M1 and the relative changes in normalized tendon stiffness from M1 to M4 in the intervention group (*r* = 0.72, *p* = 0.028) but not in the control group (*r* = 0.58, *p* = 0.081; Fig. 5B).

Maximum patellar tendon elongation did not differ between the two groups at M1 (*p* = 0.386, Table 1). The control group showed a significant increase over time (*p* = 0.017), whereas the intervention group displayed no change (*p* = 0.794, Table 1). The time-by-group interaction was not significant (*p* = 0.122). The PSF of the proximal patellar tendon did not differ at M1 between the two groups (*p* = 0.219) and did not show any significant changes over time in both control (*p* = 0.907) or intervention (*p* = 0.307) group, and no time-by-group interaction (*p* = 0.507, Table 1). No effects of group, time or time-by-group interaction were found for patellar tendon resting length and lever arm (*p* > 0.05, Table 1). The number of athletes reporting patellar tendon symptoms showed no systematic change in any group (Table 1).

## Discussion

In this longitudinal study, we investigated the influence of a personalized loading program on patellar tendon strain as an indicator for muscle–tendon imbalances in adult volleyball athletes throughout a competitive season. The intervention group received a personalized exercise prescription, based on individually determined maximum patellar tendon strain. Briefly, low strains (ε_max_ < 4.5%) were interpreted as deficit in muscle strength, intermediate strains (4.5 ≤ ε_max_ < 9%) as balanced muscle–tendon unit properties and high strains (ε_max_ ≥ 9%) as deficit in tendon stiffness. As none of the athletes showed deficits in muscle strength in relation to tendon stiffness, all athletes in the intervention group received a loading protocol with a personalized load intensity to achieve 4.5–6.5% tendon strain during training (i.e., effective range for tendon adaptation). While the maximum patellar tendon strain and, hence, the frequency of athletes with high-level tendon strain (≥ 9%) increased in the control group throughout the season, both remained widely unchanged in the intervention group and athletes with higher tendon strain at M1 demonstrated substantial reductions in maximum strain. The findings indicate a more balanced adaptation between muscle and tendon over the competitive season in the intervention group, which largely confirms our first hypothesis and may reduce the risk of tendon strain-related injury. However, in contrast to our second hypothesis, there were no systematic changes in the micromorphological properties of the tendon in both groups.

The increased maximum patellar tendon strain and frequency of athletes with tendon strain ≥ 9% over the competitive season in the control group demonstrate an increase of muscle–tendon imbalances due to the sport-specific loading in training and competition. This phenomenon implies an increase in the mechanical demand on the tendon, given that the ultimate strain appears relatively consistent across various tendons (LaCroix et al. 2013). In vitro studies have highlighted the risk of repeated exposure to high tendon strains, which can increase the likelihood of tendon injuries (Wren et al. 2003; Wang et al. 2013). More recently, we found in a prospective longitudinal study that future symptomatic athletes showed higher levels of patellar tendon strains during MVCs before developing symptoms compared to athletes who remained asymptomatic (Mersmann et al. 2023) and that the injury risk was estimated to be 2.3 times higher for athletes with a maximum patellar tendon strain ≥ 9%. In the present study, the fraction of athletes with strains above this threshold increased markedly in the control group from 18 to 80% (Fig. 3C). Therefore, an increasing number of athletes in the control group may have been subjected to a higher risk of overuse injury over the course of the season. Conversely, in the intervention group, the specific personalized tendon loading prescription prevented a systematic increase in tendon strain and even led to a reduction of maximum tendon strain in those participants with the most pronounced muscle–tendon imbalances at M1 (Fig. 5A). Further, the individual strain fluctuations, quantified in the present study using the individual residuals from the LMM at each assessment, showed a decrease in the intervention group. Given the absence of a significant in- or decrease in maximum tendon strain, this suggests a more uniform adaptation between muscle strength and tendon stiffness, which leads to more stable tendon strain over time, can prevent episodes of critically increased levels of tendon strain and may cause a higher consistency in the mechanical demand on the tendon. Therefore, the identification of muscle–tendon imbalances and a personalized exercise prescription may provide new opportunities for the prevention of strain-induced tendon overuse injuries not only in elite adolescent (Domroes et al. 2024) but also in adult athletes.

**Fig. 6:**
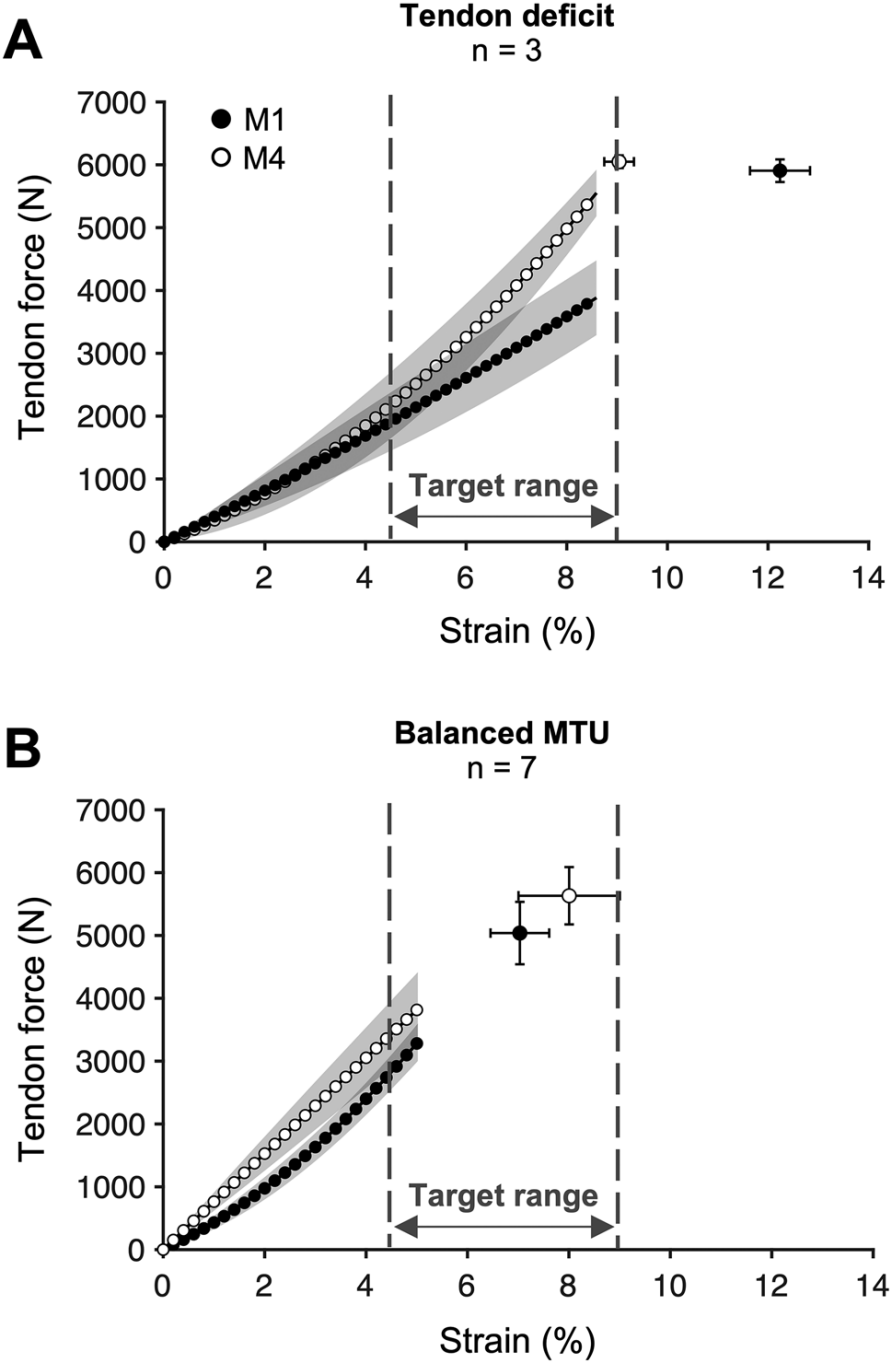
Patellar tendon force–strain relationship before (M1: black) and after (M4: white) the intervention period in the athletes participating in the personalized exercise program. Athletes with marked deficits in tendon stiffness in M1 (**A**) demonstrated a clear increase in normalized stiffness (i.e., slope of the force–strain relationship) and no increase in the maximum force applied to the tendon, which reduced maximum tendon strain to the upper margin of the proposed target range (i.e., balanced muscle strength and tendon stiffness). In athletes with a balanced muscle–tendon unit (MTU) at M1 (**B**), tendon strain remained within the target ranged despite an increase in the force applied to the tendon. Note that no statistical analysis was applied to these sub-groups due to the small sample size

In vivo, the operating tendon strain during muscle contractions is the result of the capacity of the respective muscle(s) to generate force and the normalized stiffness of the tendon. In this study, the control group demonstrated a significant increase in muscle strength of about 8.2% over the course of the season. The strength gains could be related to the increased sport-specific loading volume that typically follows the onset of a competitive season (Clemente et al. 2020) and the complementary athletic training, which has the potential to increase strength in trained athletes during a season (Sánchez Moreno et al. 2017). The sport-specific plyometric loading, however, is apparently not a particularly effective stimulus for tendon adaptation (Bohm et al. 2014; Burgess et al. 2007), which may relate to the combination of high strain rates and low strain durations. Recent in vitro observations suggest that high strain rates reduce the fraction of tenocytes responding to a given magnitude of strain (Passini et al. 2021). Accordingly, the control group did not show a significant increase in tendon stiffness to compensate for the improvements in muscle strength. In the intervention group, there was only a tendency for an increase in muscle strength (*p* = 0.064) during the season; however, the average increase was similar to the control group (8.2% for the control and 6.8% for the intervention group with no time-by-group interaction). On average 38% of the athletes in the intervention group showed maximum strain values ≥ 9% during the season and, therefore, the personalized loads to achieve the target tendon strain of 5.5% were of low to moderate relative intensity (42–57% MVC). The reduced loading intensity prescribed for this subgroup of athletes may explain why there was no additional effect on muscle strength due to the intervention. Although we only found a tendency (*p* = 0.051) for an increase in normalized tendon stiffness over the season in the intervention group at the group level, significant relationships between maximum tendon strain at M1 and changes in tendon strain and normalized stiffness were found. Figure 6A further shows the changes in the patellar tendon force–strain relationships from M1 to M4 in the subgroup of athletes from the intervention group that had a marked deficit in tendon stiffness at baseline, illustrating a clear increase in normalized stiffness and no increase in the maximum force applied to the tendon (due to the low relative exercise intensity in this subgroup). As a result, maximum tendon strain was clearly reduced. The other subgroup—athletes who were diagnosed with a balanced relationship of muscle strength and tendon stiffness (Fig. 6B)—strain at a given force tended to decrease while the maximum applied force tended to increase and maximum tendon strain remained within the range proposed to indicate a balanced overall relationship. These findings indicate that, at the individual level, the applied personalized exercise prescription reduced imbalances especially athletes with marked deficits in tendon stiffness, while it enabled a balanced increase of muscle strength in others.

Despite the general benefits of the personalized intervention with regard to the prevention of an increase in tendon strain, some participants in the intervention group sustained elevated tendon strain levels (≥ 9%) during the season. This phenomenon might be explained by an intrinsic variability in the potential of different tendons to adapt to mechanical loading. As recent findings suggest, genetically mediated variations, especially in the activity of the mechanosensitive ion channel PIEZO1, can impact tendon adaptations to mechanical loading (Passini et al. 2021; Götschi et al. 2023). Moreover, disparities in regular training and competition loads due to position-specific demands might have added to the complexity of the relationship of loading and muscle–tendon adaptation. Lastly, it is an interesting observation that the metabolic response of tendons seems to saturate after a certain overall loading volume (Magnusson et al. 2010). Considering that sub-rupture damage progression (Ros et al. 2019; Zitnay et al. 2020) and associated catabolic responses (Lavagnino et al. 2006) may further increase with loading volume, it seems possible that the increase of acute or high overall workload in the early competitive season (Clemente et al. 2020) might lead to unfavorable net-metabolic responses in certain individuals (Magnusson et al. 2010).

In vitro studies have demonstrated that repeated high loading can impair the structural integrity of tendon tissue (Fung et al. 2009; Wang et al. 2013; Wren et al. 2003). One method to estimate the latter in vivo is through a spatial frequency analysis of tendon ultrasound images (Bashford et al. 2008; Kulig et al. 2016). Briefly, lower PSF values suggest a disorganized tendon microstructure, typically seen in tendinopathic tendons (Bashford et al. 2008; Kulig et al. 2013). In earlier studies from our group, we observed an inverse association of maximum patellar tendon strain and proximal PSF in adolescent athletes (Mersmann et al. 2021, 2019). However, in the present study, the structural integrity of the proximal patellar tendon was predominantly maintained across all participants. Despite the increase of tendon strain observed in the control group, there were no indications for systematic micromorphological changes. The different findings may be explained by changes in tendon properties that unfold from adolescence to early adulthood (Charcharis et al. 2019; Rudavsky et al. 2019), whereafter the structural composition of the tendon core stabilizes and may be less sensitive to acute changes in the mechanical demand (Zhang et al. 2020; Heinemeier et al. 2013). Therefore, it seems possible that severe structural changes—detectable by means of 2D ultrasound—only occur in chronically pathological adult tendons (Kulig et al. 2013).

In the present study, the athletes of the control and intervention group were not randomly allocated. Though there is a clear advantage in terms of feasibility and within-group distribution of players from the respective positions (e.g., setter, outside-hitter etc.), it also entails some limitations. For example, there may have been differences between groups in terms of training content other than the applied intervention (e.g., number of jumps or change-of-direction movements performed), which we were not able to control for. However, as the playing level, competition schedule, as well as the weekly hours of sport-specific and athletic training per week were the same in both groups, we are confident that the differences observed over time were related to our intervention.

Due to constraints with regard to the scheduling of the measurements, it was not possible in the present study to obtain data from both groups at the same time points within the season. These temporal differences were considered in the linear mixed model by means of a week-specific coding of the data. Nevertheless, under the assumption that specific time points in the season would be associated, for example, with particularly high or low tendon strain, this would introduce a potential bias on the slope estimates of the model. However, similar to the data of the main measurements, also the additional intermediate assessments of maximum tendon strain in the intervention group do not indicate a systematic increase of imbalances at any time point in the season in that group, as maximum tendon strain was not significantly different compared to M1 in both instances (*p* = 0.32 and 0.96 in a Bonferroni-adjusted *t*-test, respectively). Therefore, we are confident that our main conclusions hold true regardless of the differences in measurement scheduling.

To obtain a sample representative for well-trained volleyball athletes, we included symptomatic athletes in both groups, as long as they were able to participate in maximum strength testing. Nevertheless, due to potential degenerative changes of the tissue and deficits in muscle activation, the decision for an inclusion may have added to the variability of the outcomes within the groups. However, both the number of symptomatic athletes and average VISA-P scores are unable to explain the changes of tendon strain observed in each of the two groups from M1 to M4 (neither at the group or individual level). Further, since there was no correlation between the M1 PSF values (as estimate of the structural integrity of the tissue) and the changes in normalized tendon stiffness from M1-4 (*r* = 0.01, *p* = 0.97), it does not appear that the changes in tendon mechanical properties were affected by potential degenerative changes of the included tendons, which can also occur irrespective of the presence or absence of symptoms. Therefore, we are confident that the inclusion of symptomatic athletes and the variability in tendon structure did not affect our main findings and conclusions.

It should also be noted that since the drop-out in both groups, which were due to a lack of time or interest and injuries or illnesses unrelated to the study, was higher than anticipated, the statistical power of the present study may have been too low to identify some relevant moderate-to-large effects (e.g., a potential time-by-group interaction on normalized tendon stiffness). Further, in the present study, no measures of tendon cross-sectional area were obtained and, thus, potential morphological changes that contribute to the observed changes in the tendon mechanical properties remain unknown. Finally, it needs to be mentioned that also non-personalized tendon loading programs with uncontrolled magnitudes of tendon strain can elicit favorable effects on tendon mechanical and structural properties (Mersmann et al. 2021). Therefore, the additional benefit of the personalized load prescription still needs to be established.

## Conclusion

This study evaluated a new approach for personalized muscle–tendon diagnostics and loading. Deficits in either muscle strength or tendon stiffness were identified based on tendon strain and, for the first time, personalized training loads were prescribed to promote tendon adaptation in male adult athletes. The personalized training was effective in preventing an increase in patellar tendon strain over the competitive season in the intervention group and increased tendon stiffness particularly in athletes demonstrating high levels of strain at M1. The observed reduction of the fluctuations of tendon strain and the lower fraction of athletes with high-level tendon strain ≥ 9% compared to the control group may potentially contribute to injury prevention, given the increased risk of developing tendon pain associated with elevated tendon strain (Mersmann et al. 2023). Due to the individual differences in maximum tendon strain, there was a substantial variation in the relative training loads (i.e., between 42 and 90% of the MVC) to achieve an average tendon strain of 6.4% during the exercises, which is considered to lie within a particularly effective range for tendon adaptation (Arampatzis et al. 2007, 2010; Bohm et al. 2014). Such a disparity underscores the limitations of relying solely on muscle strength measurements for tendon exercise prescription, which could even lead to critical strains during exercise in individuals with marked muscle–tendon imbalances (Weidlich et al. 2023). With personalized loading, it may be possible to provide specific stimuli for load-induced tendon adaptation and reduce the risk of overuse, which can serve not only as a potent preventive measure, but also as a valuable strategy to counteract present muscle–tendon imbalances.

## Data Availability

The raw data supporting the conclusions of this article will be made available by the corresponding author, without undue reservation.

## Abbreviations

LMM: Linear mixed model
M1-4: Measurement time point 1–4
MVC: Maximum voluntary contraction
PSF: Peak spatial frequency
ROI: Region of interest
TF_max_: Maximum force applied to the tendon
VISA-P: Victorian Institute of Sport Assessment for Patellar Tendinopathy
ε_max_: Maximum tendon strain
